# Development of Poly(vinyl alcohol) Grafted Glycidyl Methacrylate/Cellulose Nanofiber Injectable Hydrogels for Meniscus Tissue Engineering

**DOI:** 10.3390/polym15214230

**Published:** 2023-10-26

**Authors:** Jiraporn Sinna, Rachasit Jeencham, Priyapat Mueangkhot, Sorasak Sophon, Pornpattara Noralak, Romtira Raksapakdee, Piya-on Numpaisal, Yupaporn Ruksakulpiwat

**Affiliations:** 1 Research Center for Biocomposite Materials for Medical Industry and Agricultural and Food Industry, Nakhon Ratchasima 30000, Thailand; 2 School of Polymer Engineering, Institute of Engineering, Suranaree University of Technology, Nakhon Ratchasima 30000, Thailand; 3 School of Orthopaedics, Institute of Medicine, Suranaree University of Technology, Nakhon Ratchasima 30000, Thailand

**Keywords:** poly (vinyl alcohol) grafted glycidyl methacrylate, cellulose nanofiber, injectable hydrogels, meniscus, tissue engineering

## Abstract

This study aimed to develop poly (vinyl alcohol) grafted glycidyl methacrylate/cellulose nanofiber (PVA-g-GMA/CNF) injectable hydrogels for meniscus tissue engineering. PVA-g-GMA is an interesting polymer for preparing cross-linking injectable hydrogels with UV radiation, but it has poor mechanical properties and low cell proliferation. In this study, CNF as a reinforcing agent was selected to improve mechanical properties and cell proliferation in PVA-g-GMA injectable hydro-gels. The effect of CNF concentration on hydrogel properties was investigated. Both PVA-g-GMA and PVA-g-GMA hydrogels incorporating 0.3, 0.5, and 0.7% (*w*/*v*) CNF can be formed by UV curing at a wavelength of 365 nm, 6 mW/cm^2^ for 10 min. All hydrogels showed substantial microporosity with interconnected tunnels, and a pore size diameter range of 3–68 µm. In addition, all hydrogels also showed high physicochemical properties, a gel fraction of 81–82%, porosity of 83–94%, water content of 73–87%, and water swelling of 272–652%. The water content and swelling of hydrogels were increased when CNF concentration increased. It is worth noting that the reduction of porosity in the hydrogels occurred with increasing CNF concentration. With increasing CNF concentration from 0.3% to 0.7% (*w*/*v*), the compressive strength and compressive modulus of the hydrogels significantly increased from 23 kPa to 127 kPa and 27 kPa to 130 kPa, respectively. All of the hydrogels were seeded with human cartilage stem/progenitor cells (CSPCs) and cultured for 14 days. PVA-g-GMA hydrogels incorporating 0.5% and 0.7% (*w*/*v*) CNF demonstrated a higher cell proliferation rate than PVA-g-GMA and PVA-g-GMA hydrogels incorporating 0.3% (*w*/*v*) CNF, as confirmed by MTT assay. At optimum formulation, 10%PVA-g-GMA/0.7%CNF injectable hydrogel met tissue engineering requirements, which showed excellent properties and significantly promoted cell proliferation, and has a great potential for meniscus tissue engineering application.

## 1. Introduction

Meniscus tears are the most common knee injuries of all ages that are caused by sports, accidents, and degeneration [1,2]. Meniscal injury may compromise meniscus functions and leads to joint degeneration or osteoarthritis (OA) [3]. Currently, arthroscopic meniscus repair is a treatment of choice for meniscus injuries. However, the meniscal repair has limited efficacy in repairing the inner white-white zone, which has poor healing ability due to a lack of blood supply, which is similar to articular cartilage [4,5,6]. Meniscus tissue engineering has been extensively studied. Its strategies are being designed to promote new meniscus tissue in the inner white-white zone of the meniscus. Tissue engineering consists of three general components: scaffolds for cell support and transplantation, cells that can create a functional matrix, and bioactive factors that support and regulate cellular activity [7]. There are many commercialized cell-seeded scaffolds or pure scaffolds, but they are unfavorable for arthroscopic surgery. In terms of clinical practicality, a straightforward cell-seeded scaffold technique would be more practical if it could be applied by arthroscopic technique [8,9].

Hydrogels have been widely used in many fields such as flexible sensors, actuators, smart surfaces, drug delivery, wound healing, and tissue engineering due to their unique features, adjustable mechanical properties, and different aspects of their responsive behaviors [10]. As one of the important applications, biphasic injectable hydrogel systems have received considerable interest in the development of meniscus tissue engineering by minimally invasive approaches. Their properties can be injected into the human meniscus in liquid phase, and form an in situ solid hydrogel after curing. Recently, commercial fibrin glue has not only been used for sealant in clinical practice, but has also been applied as a scaffold. The commercial fibrin glue consists of two separate solutions, human fibrinogen and human or bovine thrombin. In some formulations of commercial fibrin glue, aprotinin as antifibrinolytic is incorporated in the thrombin solution [11]. The combination of the fibrinogen solution and thrombin will become a solid gel within a few minutes. However, using a fibrin glue as a scaffold is limited due to poor mechanical properties and a risk of human–animal immunological reaction [12]. To overcome this limitation, many researchers have proposed an artificial fibrin glue from biomaterials such as nanohydroxyapatite/poly (L-glutamic acid)-dextran, stearyl methacrylate/silk fibroin, and genipin cross-linked gelatin. These materials showed higher mechanical properties than commercial fibrin glue. However, these artificial fibrin gels still have lower mechanical properties compared to the requirements for meniscus tissue engineering scaffolds [13,14,15].

Poly (vinyl alcohol) grafted glycidyl methacrylate (PVA-g-GMA) is an interesting biodegradable synthetic polymer which has been applied in various biomedical applications. The benefits of PVA-g-GMA injectable hydrogel is water insolubility, short curing time using UV light, high hydrophilicity, good biodegradability, and low toxicity. In a previous report, PVA-g-GMA at various GMA concentrations (0.025 to 0.250 M) has been synthesized [16]. A cross-linked PVA-g-GMA hydrogel using 2-hydroxy-1-(4-(hydroxy ethoxy) phenyl)-2-methyl-1-propanone (Irgacure 2959) was successfully prepared by UV curing at 365 nm within 3 s to 5 min, and showed low cytotoxicity at GMA concentrations of 0.07 to 0.09 M [16]. The PVA-g-GMA hydrogel with 0.09 M GMA after immersion in phosphate-buffered saline (PBS) at 37 °C for 21 days gradually degraded by a weight loss of 35%. However, PVA-g-GMA showed some disadvantages such as poor compressive strength, and poor cell adhesion and proliferation [17,18].

Combined biomaterials or composite polymers is another option to improve material properties. In our work, cellulose nanofiber (CNF), obtained from the extraction of cassava pulp (CP), is used as a reinforcing agent. CNF possess excellent mechanical performance, biocompatibility, and nontoxicity. In addition, CNF provides a microenvironment similar to the extracellular matrix of the meniscus, which can promote cell viability and proliferation [19]. Currently, many studies support the advantage of CNFs as reinforcing agents, such as chitosan, sodium alginate, and gelatin for tissue engineering applications [20,21,22]. Moreover, many studies showed that CNF enhanced cell adhesion and proliferation without any toxic effects [23,24,25].

**Figure 1:**
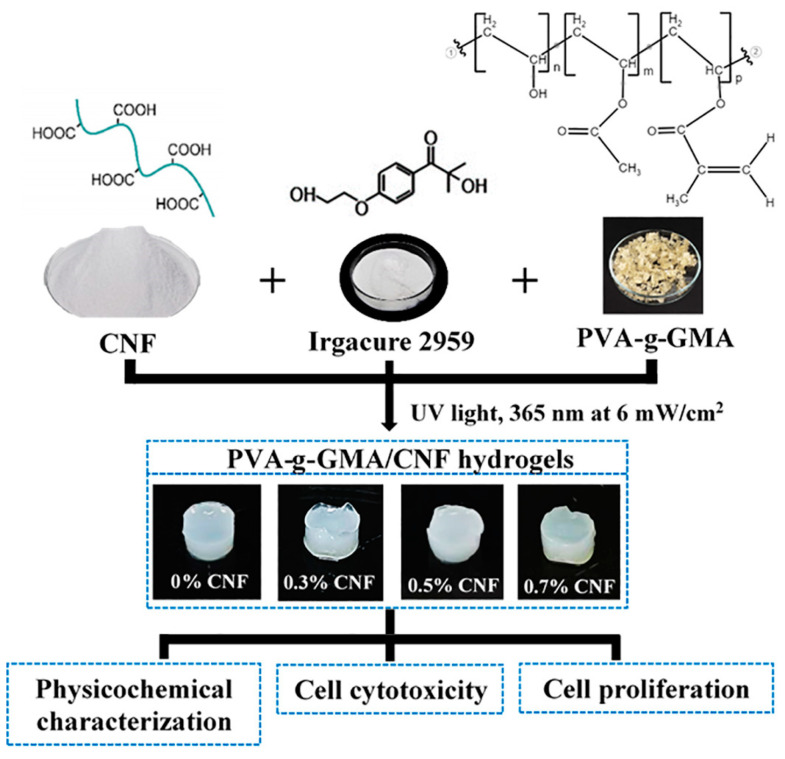
Diagram of the experimental design.

Although there are some reports on the development of PVA-g-GMA-based injectable hydrogel systems for other applications, using high UV intensity and high concentrations of GMA can be toxic to cells [16,26,27,28,29,30]. Moreover, these reported PVA-g-GMA-based injectable hydrogels have still not met the required mechanical properties for meniscus tissue engineering. With respect to the aforementioned reasons, our study aims to develop CNF-reinforced PVA-g-GMA injectable hydrogels, which are appropriate for meniscus tissue engineering. The properties, morphology, chemical interaction, pore size diameter, gel fraction, porosity, water content, water swelling, and mechanical properties were investigated. In addition, the hydrogels were seeded with human cartilage stem/progenitor cells (CSPCs) and studied for cytotoxicity and cell proliferation. The diagram of the experimental design is shown in Figure 1.

## 2. Materials and Methods

### 2.1. Materials

Poly (vinyl alcohol) (PVA, MW 13,000–23,000 g/mol, 87–89% hydrolyzed), Glycidyl methacrylate (GMA, 97%), N,N,N′,N′-Tetramethyl-ethylenediamine (TEMED, 99%), 2-Hydroxy-4′-(2-hydroxyethoxy)-2-methylpropiophenone (Irgacure 2959), Dulbecco’s modified Eagle’s medium (DMEM)-high glucose, PLTGold^®^ Human platelet lysate and L-glutamax, and MTT (3-[4,5-dimethylthiazol-2-yl]-2,5 diphenyl tetrazolium bromide) were purchased from Sigma-Aldrich Corporation (St. Louis, MI, USA). Cassava pulp was obtained from Sanguan Wongse Industries Co., Ltd. (Nakhon Ratchasima, Thailand). Sodium chlorite (NaClO_2_, technical grade, 80%) was purchased from Thermo Fisher Scientific Inc. (Waltham, MA, USA). Glacial acetic acid and dimethyl sulfoxide (DMSO) were purchased from RCI Labscan Co., Ltd. (Bangkok, Thailand). Acetone was purchased from Carlo Erba Reagent Reagents (Val-de-Reuil, France). Sodium hydroxide (NaOH, 98%) was supplied by AGC Chemicals (Thailand) Co., Ltd. (Bangkok, Thailand). Deionized water (DI) was used in this study.

### 2.2. Preparation of CNF

Cassava pulp (CP) with a particle size of 150–250 µm was obtained from grinding and sieving. To remove excess moisture, CP was dried in an oven at 120 °C for 24 h, and then 200 g of dried CP was boiled in 4 L of 4 wt% NaOH solution at 80 °C for 2 h. After that, treated CP was washed with DI water until the pH of the washing was neutral. The alkali-treated CP was dried at 80 °C for 24 h. To prepare the bleaching solution, acetate buffer solution (27 g NaOH and 75 mL glacial acetic acid, diluted to 1 L of distilled water) was mixed with 1 L sodium chlorite solution (1.7% (*w*/*v*) NaClO_2_ in water). The alkali-treated CP was mixed with bleaching solution at a ratio of 1:20 under stirring at 80 °C for 6 h. The CP, after bleaching, was washed in DI water until the pH of the washing was 7. The bleached CP was dried at 80 °C for 24 h, and then ground and sieved with a particle size of 38–63 µm. The high-pressure homogenizer (Microfluidics M-110EH-30, Microfluidics International Corporation, West-wood, MA, USA) was used to prepare CNF by separating the fibril bundles of bleached CP. Briefly, 0.5% (*w*/*v*) bleached CP suspension was prepared by dispersing in 10% DMSO in DI water. The high-pressure homogenizer for homogenization of CP was conducted for producing CNF with a pressure of 25,000 psi for 15 cycles. The obtained CNF showed an average diameter of 27 nm, an average length of 1802 nm, and gave a high crystallinity of 43% [31].

### 2.3. Grafting of PVA-g-GMA

The graft modification of PVA-g-GMA was performed by the transesterification of PVA with GMA. Briefly, 5 g of PVA was added in 100 mL of DMSO solution at 60 °C under stirring until it was completely dissolved. After that, 100 mM of GMA was incorporated into the solution, and 0.17 mL of TEMED as a catalyst was then incorporated. The reaction was stirred at 60 °C for 6 h. The PVA-g-GMA solution was cooled to room temperature (RT) and precipitated with acetone. The precipitated PVA-g-GMA was dried at RT in a fume hood for 24 h, in a hot air oven at 60 °C for 48 h, and in a vacuum oven at 45 °C for 24 h.

### 2.4. Characterization of PVA-g-GMA

#### 2.4.1. Chemical Structure

The chemical structure of PVA unmodified, GMA monomer, and PVA-g-GMA were characterized by a Tensor 27 Fourier transform infrared (FTIR) spectrometer (Bruker, Billerica, MA, USA) with attenuated total reflectance (ATR-FTIR) mode measurements. The infrared spectra of hydrogel samples were recorded in the wavenumber range of 4000 to 400 cm^−1^.

#### 2.4.2. Degree of Methacrylate Substitution of PVA

The chemical structure and degree of methacrylate substitution (DS%) of PVA unmodified, GMA monomer, and PVA-g-GMA 100 mM were analyzed using the 500 MHz NMR spectrometer (Bruker Avance III™ HD 500 MHz, Rheinstetten, Germany). The samples were dissolved in 700 μL DMSO-d_6_. The degree substitution percentage (DS%) of GMA grafted onto PVA was determined with ^1^H-NMR analysis. It was determined by calculating the relative area of the characteristic peaks of PVA unmodified, GMA monomer, and PVA-g-GMA 100 mM, which were quantified using Bruker Topspin software version 4.1.3. Its value was examined using Equation (1) [28].

(1) $$\mathrm{DS}\%=\frac{(\mathrm{GMA}(\mathrm{CH}2)/2)}{\mathrm{PVA}\left(\mathrm{OH}\right)+(\mathrm{GMA}(\mathrm{CH}2)/2)}\;\;\;\;\;\times 100$$

#### 2.4.3. Solubility of PVA-g-GMA

To find the optimum PVA-g-GMA concentration for injectable hydrogel preparation, the effect of PVA-g-GMA concentration on solubility was investigated. PVA-g-GMA with various concentrations of 5, 10, and 15% (*w*/*v*) was dissolved in 10% DMSO in DI water at 60 °C for 48 h. The solubility of PVA-g-GMA was then determined. The PVA-g-GMA was dried at 60 °C for 24 h (initiator weight, WI), and the undissolved PVA-g-GMA was weighed (undissolved weight, WU). The solubility of PVA-g-GMA was calculated using Equation (2).

(2) $$\mathrm{Solubility}\;(g/\mathrm{mL})=\frac{WI\left(g\right)-WU(g)}{Volume\;of\;solvent\;(mL)}\;\text{×}\;100$$

### 2.5. Preparation of PVA-g-GMA/CNF Injectable Hydrogels

To find the optimum UV radiation time of PVA-g-GMA injectable hydrogels, the effect of UV radiation time on gelation and gel fraction was investigated. The optimum PVA-g-GMA concentration was dissolved in 10% DMSO in DI water at 60 °C for 48 h. After that, 0.3% (*w*/*v*) IR2959 was mixed into PVA-g-GMA solution at 60 °C using a magnetic stirrer at 200 rpm for 30 min. The PVA-g-GMA solution was injected into the mold with a thickness of 2 mm, and cured with a UV intensity of 6 mW/cm^2^ at a wavelength of 365 nm at various times of 5, 10, and 15 min. The optimum UV radiation time for preparing PVA-g-GMA injectable hydrogels was investigated by gel fraction characterization. The concentrations of CNF were varied at 0, 0.3, 0.5, and 0.7% (*w*/*v*). The optimum PVA-g-GMA concentration was dissolved in CNF suspension at 60 °C using a magnetic stirrer at 200 rpm for 48 h. After that, 0.3% (*w*/*v*) IR2959 was incorporated into PVA-g-GMA solution at 60 °C under stirring for 30 min. The PVA-g-GMA/CNF liquid mixture was pipetted into the mold and cured by UV light at 365 nm, 6 mW/cm^2^ at optimum UV radiation time. After that, the properties of the hydrogels and cell viability assessments were determined.

### 2.6. Characterization of Injectable Hydrogel

#### 2.6.1. Morphology and Pore Size

FE-SEM (Carl Zeiss AURIGA^®^, Thuringia, Germany) was used to observe the morphology and pore size diameter of hydrogels. Before the investigation, the lyophilized hydrogel cross-sections were coated with a thin layer of gold. The pore size diameter of 100 random pores in the hydrogel samples was measured using ImageJ software version 1.53t (Wayne Rasband NIH, Washington, DC, USA). In addition, the distribution of pore size diameter was plotted using OriginLab software (OriginPro^®^ 2021b, OriginLab Corporation, Northampton, MA, USA).

#### 2.6.2. Porosity

Porosity is the percentage of void space volume in the hydrogel. Generally, the hydrogel in the physical condition is a hydrated three-dimensional structure. This implies that the liquid infiltrates and attaches to the pore of a hydrogel. Therefore, the porosity of the space volume of a hydrogel in the physical condition is the porosity of completely hydrated hydrogel. For hydrogel porosity assessment, hydrated hydrogel needs to be lyophilized before measuring the hydrogel volume for dehydration without hydrogel deformation. Ethanol was selected as a displacement liquid for the soaking solution because it could diffuse through the porous scaffolds without changing the shrinkage. The porosity of the samples was investigated by the fluid displacement method. The lyophilized samples were immersed in 5 mL ethanol (V_1_) for 10 min. The total volume of sample and ethanol (V_2_) was collected. The ethanol-infused sample was removed, and the residual volume of ethanol (V_3_) was recorded. The porosity was calculated using Equation (3). The sample number was six per group (*n* = 6).

(3) $$\mathrm{Porosity}\;(\%)=\frac{V_{1}-V_{3}}{V_{2}-V_{3}}\;\text{×}\;100\;\;\;$$

#### 2.6.3. Gel Fraction

Hydrogel samples were dried in an oven at 40 °C for 24 h and then weighed (W_0_). Dried samples were soaked in DI water at 37 °C for 24 h and dried at 40 °C for 24 h (W_1_). The gel fraction of hydrogel samples was calculated as shown in Equation (4). The sample number was six per group (*n* = 6).

(4) $$\mathrm{Gel}\;\mathrm{fraction}\;(\%)=\frac{W_{1}}{W_{0}}\;\text{×}\;100$$

#### 2.6.4. Water Content and Swelling

The hydrogel sample was soaked in DI water at 37 °C for 24 h and then the wet weight (W_w_) was recorded. The wet sample was dried in an oven at 40 °C for 24 h and then the dry weight (W_d_) was measured. The water content and water swelling of the sample were calculated using Equations (5) and (6), respectively. The sample number was six per group (*n* = 6).

(5) $$\mathrm{Water}\;\mathrm{content}\;(\%)=\frac{\left(W_{w}-W_{d}\right)}{W_{w}}\;\text{×}\;100\;\;$$

(6) $$\mathrm{Water}\;\mathrm{swelling}\;(\%)=\frac{\left(W_{w}-W_{d}\right)}{W_{d}}\;\text{×}\;100\;\;\;$$

#### 2.6.5. Compressive Properties

The biomechanical behavior of meniscal tissue in compression is typically investigated. The rate of speed in the compression test of meniscal tissue mimicking the speed rate in compression of the knee joint is between 2 and 10 mm/min [32,33]. From the condition in the reference literature, we designed the condition of the compressive test near those established by Shadi, M. et al. (2022) and Xu, Z. et al. (2021) [34,35]. They used the testing condition of a speed of 3.5 mm/min and a pressure distance of 80% strain, respectively. In this study, a TA.XT-PLUS texture analyzer (Texture Technologies Corp., London, UK) was used to determine the compressive properties of the hydrogel samples using a load cell of 5 kg, with a testing condition of a speed of 3 mm/min and a pressure distance of 80% strain. The sample number was six per group (*n* = 6).

#### 2.6.6. Chemical Structure and Interaction

The chemical structure and interaction of CNF and PVA-g-GMA in the hydrogel composites were identified using a Tensor 27 FTIR spectrometer (Bruker, Billerica, MA, USA). The FTIR spectrum of hydrogel samples was recorded in the range of 4000 to 400 cm^−1^.

### 2.7. In Vitro Cell Cytotoxicity

The cell cytotoxicity of hydrogel samples was preliminary studied by the extract dilution method according to ISO standard 10993-5: biological evaluation of medical devices [36]. To prevent contamination, the hydrogel sample preparation and cytotoxicity testing were conducted in a laminar flow cabinet. The autoclaved liquid samples were pipetted into a 96-well culture plate and then irradiated with UV light at 365 nm, 6 mW/cm^2^ for 10 min to form hydrogels. Cytotoxic activity was determined from the viability of CSPCs after incubating in the extracted medium of the hydrogels for 24 h. The CSPCs were obtained according to the following protocol, which was proven for purity using flow cytometry analysis. Under the approval of the institutional review board, cells from knee articular cartilage of the donors were isolated and cultured. The purity of the cultured cells was confirmed by positive marker expression (CD73, CD90, and CD105) of more than 95%, and negative marker expression (CD34, CD45, and HLA-DR) of less than 2%. The 3-(4,5-dimethylthiazol-2-yl)-2,5 diphenyl tetrazolium bromide (MTT) assay was used as a protocol for cell viability assessment. The extracted medium of the samples was obtained from 24 h immersion of samples in cell culture media (5% PLTGold^®^ Human platelet lysate, 1% L-glutamax, 1% antibiotic and Dulbecco’s modified Eagle’s medium (DMEM)-high glucose). The CSPCs (2.5 × 10^4^ cells) were seeded onto a 96-well culture plate which contained 200 µL of culture media, and then incubated at 37 °C for 24 h in 5% CO_2_. After that, 200 µL of extracted media was used instead of the old culture media in the cell-seeded 96-well culture plates, and then incubated at 37 °C for 24 h in 5% CO_2_. The cell viability of CSPCs after incubating in the extracted medium was investigated by the MTT assay. Briefly, the cells were gently washed with PBS, and 100 µL MTT solution was pipetted into cell-seeded 96-well culture plates and incubated for 2 h for MTT. After decanting the MTT solution, 100 µL DMSO was pipetted to dissolve the extracted formazan crystals for 10 min. The extracted formazan medium was measured for absorbance at the wavelength of 570 nm using an Infinite M200 Pro microplate reader (Tecan Group Ltd., Männedorf, Switzerland). The cell viability of hydrogel samples was compared to cells cultured in a medium without extracted hydrogel (control). The sample number was three per group (*n* = 3). The percentage of cell viability was calculated as shown in Equation (7). For the extracted formazan absorbance of each sample at 570 nm (OD_570A_), divide by the extracted formazan absorbance for control at 570 nm (OD_570B_) and multiply by 100.

(7) $$\mathrm{Cell}\;\mathrm{viability}\;(\%)=\frac{{\mathrm{OD}}_{570A}}{{\mathrm{OD}}_{570B}}\;\text{×}\;100\;\;$$

### 2.8. Cell Proliferation

Cell proliferation in hydrogels was investigated by CSPC viability after culturing on hydrogel samples for 1 and 14 days. The cell viability was investigated by the MTT assay. To prevent contamination, the hydrogel sample preparation and cell viability assay were performed in a laminar flow cabinet. The autoclaved liquid samples were pipetted into a 96-well culture plate and then irradiated with UV light at 365 nm, 6 mW/cm^2^ for 10 min to form hydrogels. The CSPCs (2.5 × 10^4^ cells) were seeded onto hydrogel which contained 200 µL of culture media, and then incubated at 37 °C in 5% CO_2_ for 1 and 14 days. The CSPC viability on hydrogel samples with cell culture for 1 and 14 days was determined by the MTT assay. The sample number was three per group (*n* = 3), and the protocols are described in Section 2.7, in vitro cell cytotoxicity.

### 2.9. Statistical Analysis

The quantitative data are indicated as mean ± standard deviation. The physicochemical properties (gel fraction, porosity, water content, water swelling, and compressive properties) were performed on six replicate samples (*n =* 6), whereas cell cytotoxicity and cell proliferation were conducted on three replicate samples (*n* = 3). For all comparison groups, statistical differences were analyzed with one-way ANOVA and paired t-test, followed by Tukey’s post hoc comparison test, and a *p* < 0.05 was considered statistically significant.

## 3. Results and Discussions

### 3.1. Characterization of PVA-g-GMA

#### 3.1.1. Chemical Structure

The spectra of PVA unmodified, GMA monomer, and PVA-g-GMA 100 mM are shown in Figure S1. The characteristic peak of unmodified PVA showed transmittance bands at 842, 1089, 1567, 1707, 2940, 2910, and 3298 cm^−1^, which referred to the vibrations of =C-H stretching, C-O stretching, C=C stretching, C=O (ester group), CH_2_ symmetric stretching, C-H stretching, and O-H stretching, respectively. GMA monomer showed a peak at 1255 cm^−1^ breathing, 908 cm^−1^ asymmetric deformation, and 843 cm^−1^ symmetrical deformation, which was the characteristic vibration of epoxy groups. FTIR peaks at 1717 cm^−1^ and 1636 cm^−1^ were attributed to the carbonyl group (C=O) and C=C group, respectively. In addition, the peak at 1159 cm^−1^ corresponded to the C-O stretching of the ester group. The distinctive bands of PVA and GMA on PVA-g-GMA, such as the bending of C=O at 1710 cm^−1^ and C=C at 1634 cm^−1^, out-of-plan bending vibration of R_2_C=CH_2_ at 949 cm^−1^, and stretching vibration of the C-O group at 1175 cm^−1^, could be found in the spectra of PVA-g-GMA. In addition, without the bands at 1255 cm^−1^ (for breathing), 908 cm^−1^ (for asymmetric deformation), and 843 cm^−1^ (for symmetrical deformation), the epoxy ring of GMA clearly implies that transesterification was the mechanism via which GMA and PVA reacted [28].

#### 3.1.2. Degree of Methacrylate Substitution in PVA

^1^H-NMR spectra were used to determine the number of methacrylate groups in PVA-g-GMA, as well as for identifying the reaction of the mechanism of transesterification. The ^1^H-NMR peak of unmodified PVA, GMA monomer, and PVA-g-GMA at 100 mM are shown in Figure S2. The solvents of DMSO-d_6_ in the polymer structures exhibited sharp peaks at chemical shifts (δ) of 2.5 ppm. In the spectra of unmodified PVA, the characteristic proton peaks of (CH_2_-CH-OH) were at δ = 4.3–4.7 ppm, (CH_2_-CH-OH) at δ = 3.7–4.0 ppm, and (CH_2_-CH-OH) at δ = 1.4 ppm. In the spectrum of PVA-g-GMA, in addition to the peaks of unmodified PVA, new peaks appeared belonging to the protons of the vinyl group (-OCO-C (CH_3_) = CH_2_), which appeared at δ = 5.6 and 6.0 ppm and were attributed to the distinctive double bond of the methacrylate group, proving the methacrylate group was effectively grafted onto the pendant hydroxyl groups of unmodified PVA, the CH group of GMA (-CH- of O-methacrylate group) at δ = 5.2 ppm, and the methyl group (CH_3_-C=C) of GMA unit appeared at δ = 1.9 ppm [28]. The percentage of GMA functional group, or degree of methacrylate substitution, DS% of the PVA-g-GMA 100 mM was also calculated with Equation (1). The DS% of GMA grafted on PVA was 12.06%.

#### 3.1.3. Solubility of PVA-g-GMA

From the preparation of PVA-g-GMA solution, PVA-g-GMA at concentrations of 10%, 15%, and 20% (*w*/*v*) were dissolved in 10% (*v*/*v*) DMSO in DI water. The 10% (*w*/*v*) PVA-g-GMA was completely soluble in the DMSO–DI water mixture solution. On the other hand, PVA-g-GMA at 15% and 20% (*w*/*v*) concentrations were not completely soluble in DMSO/DI water mixture solution. Their solubility values were not different, 0.109 g/mL (10.90% *w*/*v*) and 0.107 g/mL (10.70% (*w*/*v*), respectively. This indicated that 10% (*w*/*v*) PVA-g-GMA is an appropriate concentration to further study the effect of UV radiation time on gelation and gel fraction of hydrogels.

### 3.2. Effect of UV Radiation Time on Gelation and Gel Fraction of Hydrogel

The 10%PVA-g-GMA hydrogel can be formed by curing with UV at a wavelength of 365 nm, 6 mW/cm^2^ for 5, 10, and 15 min. The sample number was six per group (*n* = 6) and the optimal UV radiation time was investigated. The optimum UV radiation time for preparing PVA-g-GMA injectable hydrogels was investigated by gel fraction characterization. The gel fraction of PVA-g-GMA hydrogels with various UV radiation times is shown in Table 1. Generally, the gel fraction of tissue engineering requirements for hydrogels should be ≥80% [37]. The hydrogel at UV curing for 5 min showed a low gel fraction of 67%, while the hydrogel at UV curing for 10 and 15 min met the requirement, which showed a gel fraction of more than 80%. When UV irradiates, the photoinitiator (Irgacure 2959) absorbs UV and converts this light energy into chemical energy in the form of free radicals, which subsequently initiate cross-linking by polymerizing the methacrylate units of PVA-g-GMA, and forms as a hydrogel. This indicates that prolonging the irradiation time (10 and 15 min) could allow for increasing the number of free radicals, which results in a high gel fraction or a high degree of cross-linking of PVA-g-GMA hydrogel [16]. Consequently, the optimum UV radiation time strongly affects the gel fraction of PVA-g-GMA hydrogels.

### 3.3. Characterization of CNF/PVA-g-GMA Injectable Hydrogels

In this study, we studied the possibility of developing PVA-g-GMA/CNF injectable hydrogels for applying the materials for meniscus tissue engineering. The purpose of incorporating CNF into PVA-g-GMA injectable hydrogel was to improve mechanical properties and promote cell proliferation. Thus, the effect of CNF on hydrogel properties and preliminary studies of cell culture (cytotoxicity and cell proliferation by MTT assay) were investigated.

#### 3.3.1. Physicochemical Characterization

##### Appearances

**Figure 2:**
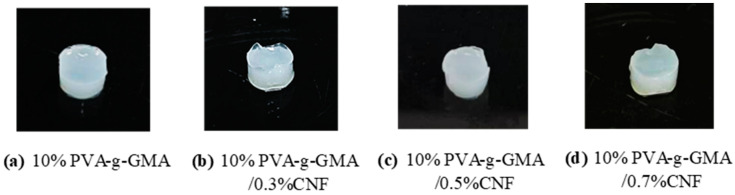
Appearances of injectable hydrogels (**a**) 10%PVA-g-GMA, (**b**) 10%PVA-g-GMA/0.3%CNF, (**c**) 10%PVA-g-GMA/0.5%CNF, (**d**) 10%PVA-g-GMA/0.7%CNF.

All PVA-g-GMA/CNF hydrogels at CNF concentrations from 0 to 0.7% (*w*/*v*) with a thickness of 2 mm were successfully generated by UV exposure for 10 min, which showed a uniformly turbid white colour and were nonbrittle, as shown in Figure 2.

##### Morphology, Pore Size Diameter, and Porosity

**Figure 3:**
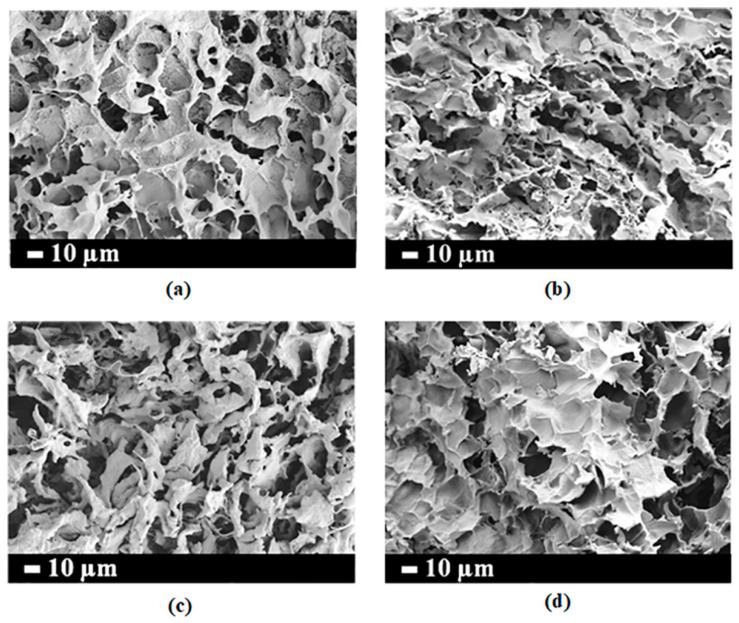
FE-SEM micrographs and pore size diameter of (**a**) 10%PVA-g-GMA, (**b**) 10%PVA-g-GMA/0.3%CNF, (**c**) 10%PVA-g-GMA/0.5%CNF, (**d**) 10%PVA-g-GMA/0.7%CNF injectable hydrogels.

FE-SEM images of a cross-section of lyophilized hydrogels at various CNF concentrations are shown in Figure 3. All hydrogels were substantially microporous with interconnected tunnels (Figure 3) and a pore size diameter range of 3–68 µm (Table 2 and Figure S3). In addition, the porosity of all injectable hydrogels was 83–94% (Table 2). It is worth noting that the reduction of porosity in the hydrogels occurred with increasing CNF concentration because CNF may be spread in the polymeric structure. However, the porosity and pore sizes of all hydrogels meet the requirements of tissue engineering, which exhibited pore sizes ranging from 5 to 350 µm and a porosity of ≥55% [38]. This suggests that all hydrogels showed suitable pore sizes, which are essential for cell growth and proliferation.

##### Gel Fraction, Water Content, and Swelling

Gel fraction is the cross-linking degree of cross-linked hydrogel after water immersion. The percentage of undissolved fractions indicates the percentage of cross-links formed. Generally, the gel fraction requirement of hydrogels should be ≥80% [37]. From the results in Table 2, the gel fraction of all hydrogels exceeds 80%, meeting the requirement, and suggesting that the hydrogels are hydrophilic polymer networks that retain their integrity in an aqueous environment.

Water content and swelling are important characteristics of tissue engineering materials that directly influence cellular nutrient transport. The water content and swelling requirements in tissue engineering should be ≥50% and 100%, respectively [39]. PVA-g-GMA and PVA-g-GMA/CNF injectable hydrogels met the property requirements, with high water content (73–87%) and water swelling (272–652%) (Table 2). Especially, water content and swelling of hydrogels were increased when CNF concentration increased. These results were similar to those obtained by Baghbadorani, N.B. et al. (2020). They found that the incorporation of CNF significantly increased the water content and swelling of starch graft poly (acrylic acid) hydrogel [40].

##### Compressive Properties

**Figure 4:**
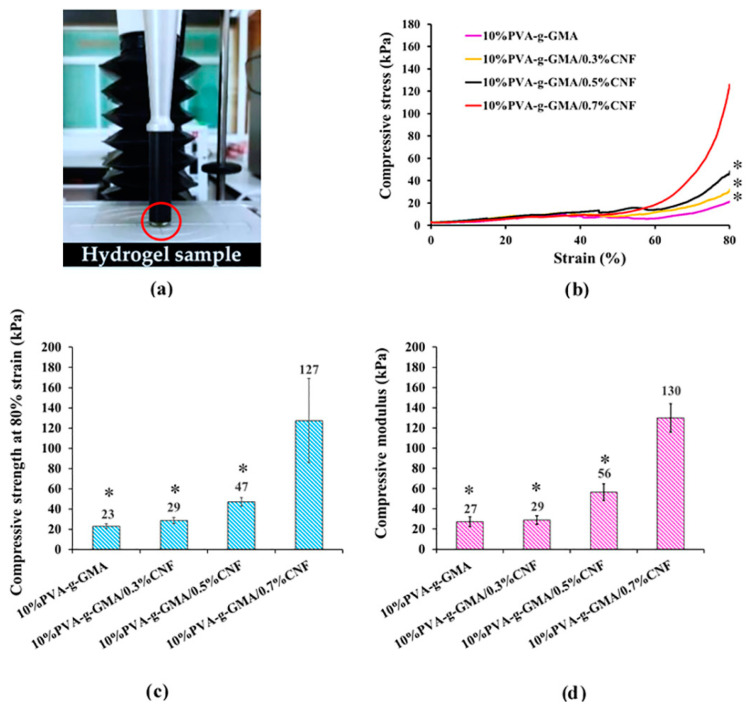
(**a**) Compressive characterization, (**b**) compressive stress–strain curve, (**c**) compressive strength at 80% strain, (**d**) compressive modulus of PVA-g-GMA and PVA-g-GMA/CNF injectable hydrogels (*n* = 6, * *p* < 0.05 compared with 10%PVA-g-GMA/0.7%CNF). The red circle indicates hydrogel sample.

To create a high-performance injectable hydrogel for tissue-engineered meniscus, the compressive properties that withstand physiological stresses are of paramount importance to investigate. The stress–strain curves of injectable hydrogels at different CNF concentrations are shown in Figure 4b. The stress–stain behavior of PVA-g-GMA injectable hydrogels indicated soft and flexible materials. To compare the effect of CNF concentrations on the compressive properties, their compressive strength and compressive modulus are shown in Figure 4c,d, respectively. The unreinforced hydrogel (10%PVA-g-GMA) showed the lowest compressive properties, which showed a compressive strength of 23 kPa and a compressive modulus of 27 kPa. As CNF concentration increased from 0.3% to 0.7% (*w*/*v*), the compressive strength and compressive modulus of the hydrogels increased from 29 kPa to 127 kPa and 29 kPa to 130 kPa, respectively. Unfortunately, the compressive properties of PVA-g-GMA hydrogel and PVA-g-GMA hydrogel incorporating 0.3% and 0.5% (*w*/*v*) of CNF showed lower compressive properties than scaffold requirement for meniscus tissue engineering (Table 3). Especially, the 10%PVA-g-GMA/0.7%CNF hydrogel met the scaffold requirement for meniscus tissue engineering, which showed good compressive strength (127 kPa) and compressive modulus (130 kPa), as shown in Table 3. Many studies have reported the compressive properties of injectable hydrogel for orthopedic and meniscus tissue engineering from nanohydroxyapatite/poly (L-glutamic acid)-dextran, stearyl methacrylate/silk fibroin, genipin cross-linked gelatin, and fibrin glue, but their compressive properties lacked the scaffold requirement for meniscus tissue engineering (Table 3). Furthermore, 10%PVA-g-GMA/0.7%CNF hydrogel showed remarkably higher mechanical properties than other hydrogels (Table 3), which confirms the mechanical enhancement by containing CNF in the hydrogel. 

##### Chemical Structure and Interaction

**Figure 5:**
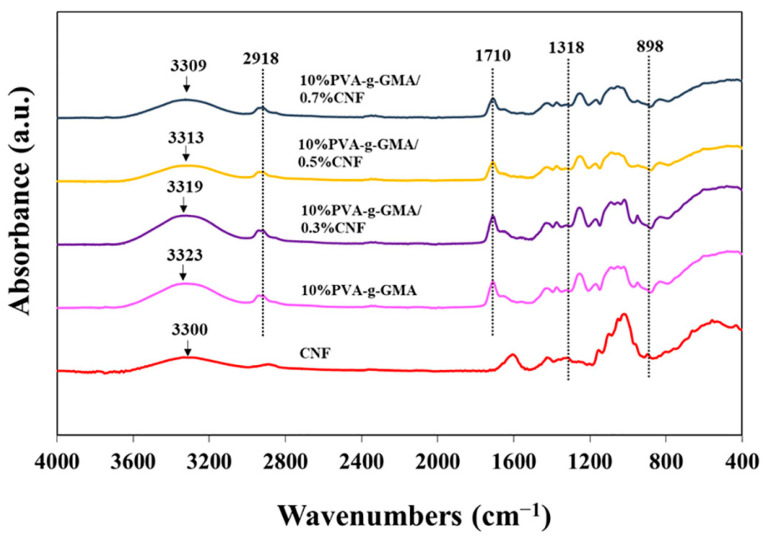
FTIR spectra of CNF, PVA-g-GMA, and PVA-g-GMA/CNF injectable hydrogels.

FTIR spectra of CNF and injectable hydrogels are shown in Figure 5. The characteristic absorption band of CNF shows the peak of OH stretching at 3300 cm^−1^. This peak corresponded to intermolecular hydrogen bonds of cellulose [42]. The chemical structure of the cellulose components was discovered at characteristic peaks of CNF, 1318 cm^−1^ and 898 cm^−1^, which were attributed to CH_2_ wag, and beta glycosidic linkages of the glucose ring [43]. The characteristic absorption band of PVA-g-GMA shows peaks at 3323, 2918, and 1710 cm^−1^, which is attributed to the vibrations of OH stretching, C-H stretching, and C=O (ester group), respectively. Characteristic peaks for CNF and PVA-g-GMA can be observed in the PVA-g-GMA/CNF hydrogel, which verify the existence of all the components in the hydrogel. Interestingly, the characteristic peak shifting of CNF and PVA-g-GMA was observed in the injectable hydrogel composite. For instance, the spectra of 10%PVA-g-GMA/0.7%CNF injectable hydrogel revealed the OH stretching peak of PVA-g-GMA shifting from 3323 to 3309 cm^−1^ because of the intermolecular H-bonding between -OH of PVA-g-GMA and -OH of CNF, suggesting good compatibility of composite hydrogels.

#### 3.3.2. Cell Cytotoxicity

**Figure 6:**
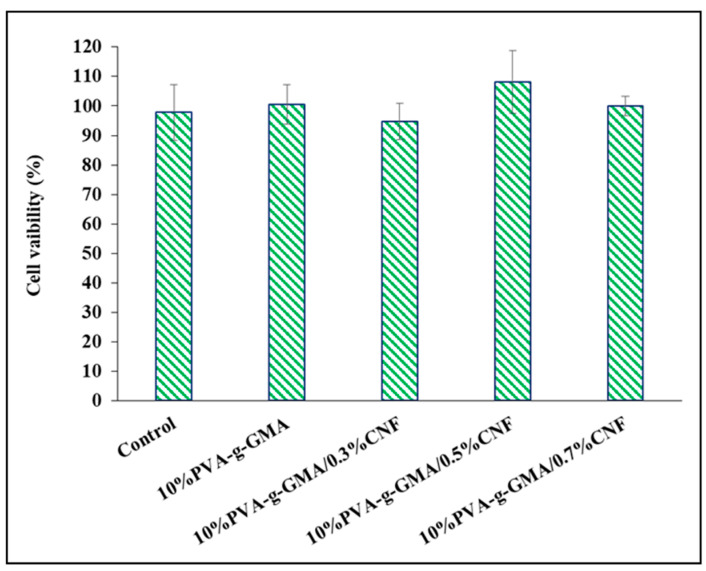
CSPC viability after incubating with hydrogel extracted culture medium (*n* = 3).

In fact, to investigate the cell viability of hydrogel by seeding the cells on the hydrogel, not only cytotoxicity, but also cell attachment are affected by the cell viability of difference hydrogel samples. As a result, we cannot tell which factor influenced the apparent value of cell viability. For the in vitro cytotoxicity assessment of medicinal products that could release toxins from exposed surfaces without other disruption factors, the extract dilution approach is currently widely employed. The CSPCs viability was measured using the MTT assay after 24 h of incubation in an extraction medium to evaluate the degree of cytotoxicity of the hydrogel. The viability of CSPCs after incubation in an extracted medium of all hydrogels was more than 95% (Figure 6), suggesting that PVA-g-GMA and PVA-g-GMA/CNF injectable hydrogels are noncytotoxic to CSPCs as determined by ISO 10993-5 (cell viability ≥ 70%).

#### 3.3.3. Cell Proliferation

**Figure 7:**
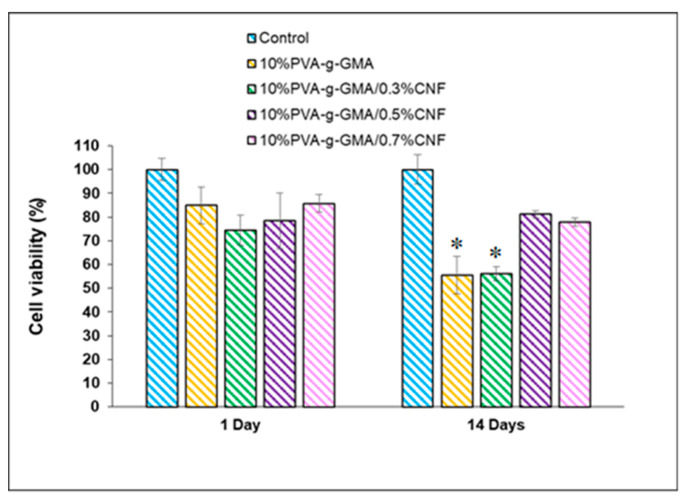
CSPCs **v**iability in injectable hydrogels after culturing for 1 and 14 days (*n* = 3, * *p* < 0.05 compared with 10%PVA-g-GMA/0.7%CNF).

**Figure 8:**
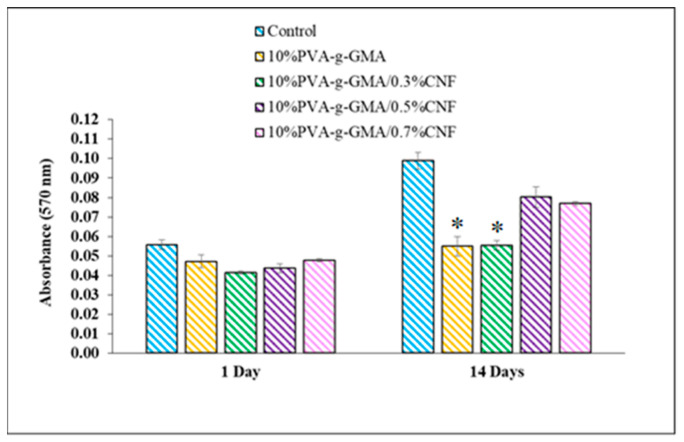
Formazan absorbance as the cell culture time progressed from 1 day to 14 days (*n* = 3, * *p* < 0.05 compared with 10%PVA-g-GMA/0.7%CNF).

The ideal biomaterial for tissue engineering scaffolds is non-cytotoxic, and enhances cell adhesion and proliferation. Cell proliferation on PVA-g-GMA hydrogels with various CNF concentrations was determined by CSPCs viability after culturing on hydrogel samples for 1 and 14 days, using the MTT assay. The cell viability of CSPCs in hydrogel with various CNF concentrations is shown in Figure 7. Figure 8 illustrates the absorbance of solubilized formazan as a function of cell proliferation following growth in injectable hydrogels for 14 days in order to comprehend the trend of cell activity. Cell viability after culturing on all hydrogel samples on day 1 was in the range of 74–86%. On Day 14, 10%PVA-g-GMA and 10%PVA-g-GMA/0.3%CNF hydrogel showed a low cell viability of 56%. In fact, these hydrogels showed noncytotoxicity, but revealed low cell viability in the proliferation assessment. This implied that these hydrogels have poor cell attachment, which leads to a decrease in cell viability in the long-term culture. In contrast, 10%PVA-g-GMA/0.5%CNF and 10%PVA-g-GMA/0.7%CNF showed high cell viability of 81 and 78%, respectively, after culturing for 14 days, implying good cell adhesion and viability in the long-term culture. In addition, PVA-g-GMA hydrogel incorporating 0.5% and 0.7% (*w*/*v*) CNF showed an increase in formazan absorbance as the cell culture time progressed from 1 day to 14 days (Figure 8). From the results, incorporating optimized CNF concentration into hydrogel led to enhance cell viability and proliferation. This may be because CNF acts as a bioadaptive ECM-mimetic cellular environment, which can promote cell viability and proliferation [19].

All of the experimental results confirm that injectable hydrogels of CNF-reinforced PVA-g-GMA represent a system alternative to existing therapeutics. These hydrogels enhance the physical and chemical properties, which ensure superior performance compared to conventional hydrogels, fibrin glue, and other developed hydrogel materials (summarized in Table 3). Especially, this CNF-reinforced PVA-g-GMA injectable hydrogel significantly promotes cell proliferation, and also shows good physicochemical properties which meet meniscus tissue engineering requirements. The results indicate that the mechanical characteristics of the injectable hydrogels and cell viability are significantly improved via incorporating CNF into the injectable hydrogels, which proves our hypothesis correct.

## 4. Conclusions

In this research, we studied the possibility of developing CNF-reinforced injectable hydrogels as biomaterials for meniscus tissue engineering applications. Thus, physicochemical properties of meniscus requirements and preliminary studies of cell culture (cytotoxicity and cell proliferation by MTT assay) were focused on. We successfully prepared injectable PVA-g-GMA/CNF hydrogels by UV curing. CNF remarkably enhanced the mechanical properties of the hydrogels, and also promoted the cell proliferation of CSPCs. At optimum formulation, 10%PVA-g-GMA/0.7%CNF showed excellent physicochemical properties and significantly promoted cell proliferation. Their properties meet tissue engineering requirements, which have a great potential for meniscus tissue engineering applications. In the next step, we will further investigate the in-depth study of quantitative analysis for gene expression, explant models, and immunohistochemistry.

## Figures and Tables

**Table 1:** Gel fraction of hydrogel with various UV radiation times (*n* = 6).

Time of UV Radiation	Gel Fraction of 10%PVA-g-GMA (%)
5 min	66.60 ± 3.50
10 min	81.37 ± 1.61
15 min	80.65 ± 1.26

**Table 2:** Pore size diameter, porosity, gel fraction, water content, and water swelling of injectable hydrogels (*n* = 6).

Formulations	Pore Size Diameter (µm)	Porosity (%)	Gel Fraction (%)	Water Content (%)	Water Swelling (%)
10%PVA-g- GMA	5–68	94.31 + 0.95	81.37 + 1.61	73.12 ± 0.77	272.30 ± 10.32
10%PVA-g-GMA/0.3%CNF	3–64	90.23 ± 0.89	82.34 ± 2.37	84.12 ± 0.69	530.73 ± 28.96
10%PVA-g-GMA/0.5%CNF	5–62	87.01 ± 1.36	81.45 ± 1.99	85.36 ± 1.15	586.66 ± 54.45
10%PVA-g-GMA/0.7%CNF	3–64	83.31 ± 1.10	82.09 ± 1.89	86.80 ± 0.61	652.19 ± 29.95

**Table 3:** The comparison in compressive properties of different hydrogels for orthopedic tissue engineering.

Materials	Compressive Strength (kPa)	Compressive Modulus (kPa)	References
Nano-hydroxyapatite/ poly (L-glutamic acid)-dextran	51.00	/	[13]
Stearyl methacrylate/silk fibroin	17.10	/	[14]
Genipin cross-linked gelatin hydrogel	70.00	300.00 ± 30.00	[15]
Fibrin glue	46.71 ± 8.87	11.98 ± 4.45	[31]
10%PVA-g-GMA	22.95 ± 2.30	27.30 ± 5.02	This work
10%PVA-g-GMA/0.3%CNF	28.79 ± 3.05	28.84 ± 4.20	
10%PVA-g-GMA/0.5%CNF	47.14 ± 4.33	56.46 ± 8.29	
10%PVA-g-GMA/0.7%CNF	127.39 ± 41.59	129.91 ± 14.07	
Meniscus scaffold requirement	≥100	≥100	[41]

Note: / means not indicated.

## Data Availability

The data presented in this study are available on request from the corresponding author.

## Supplementary Materials

The following supporting information can be downloaded at: https://www.mdpi.com/article/10.3390/polym15214230/s1.
