# Supplementary material for: Development of Poly(vinyl alcohol) Grafted Glycidyl Methacrylate/Cellulose Nanofiber Injectable Hydrogels for Meniscus Tissue Engineering
Source: Polymers (Basel). 2023 Oct 26;15(21):4230. doi: 10.3390/polym15214230 (PMC10647663; doi:10.3390/polym15214230)
Supplement: Supplementary file 1 [file polymers-15-04230-s001.zip › polymers-2672552-supplementary.pdf]

Supplementary Information

Development of Poly (vinyl alcohol) Grafted Glycidyl Methacrylate/Cellulose Nanofiber Injectable Hydrogels for Meniscus Tissue Engineering

Jiraporn Sinna [1,2], Rachasit Jeencham [1], Priyapat Mueangkhot [2], Sorasak Sophon [2], Pornpattara Noralak [2], Romtira Raksapakdee [2], Piya-on Numpaisal [1,3,*] and Yupaporn Ruksakulpiwat [1,2,*]

[1] Research Center for Biocomposite Materials for Medical Industry and Agricultural and Food Industry, Nakhon Ratchasima 30000, Thailand

[2] School of Polymer Engineering, Institute of Engineering, Suranaree University of Technology, Nakhon Ratchasima 30000, Thailand

[3] School of Orthopaedics, Institute of Medicine, Suranaree University of Technology, Nakhon Ratchasima 30000, Thailand

* Correspondence: piya-on@sut.ac.th (P.-o.N.); yupa@sut.ac.th (Y.R.)

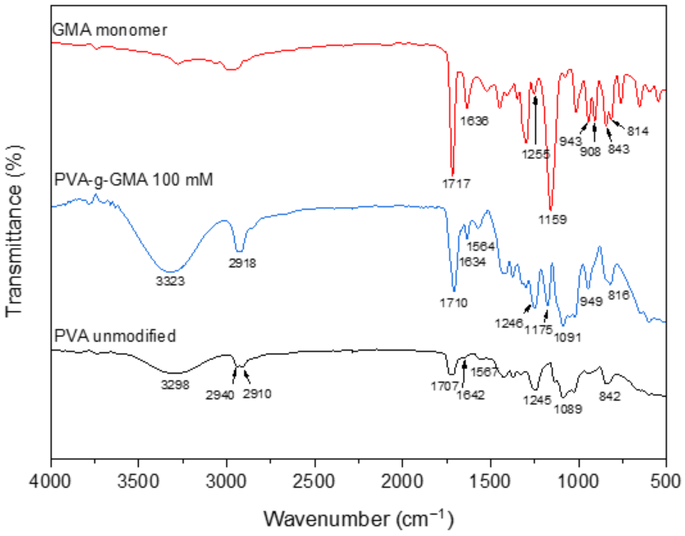

Figure S1. Comparison of the FTIR spectra of GMA monomer, PVA-g-GMA 100 mM, and PVA unmodified.

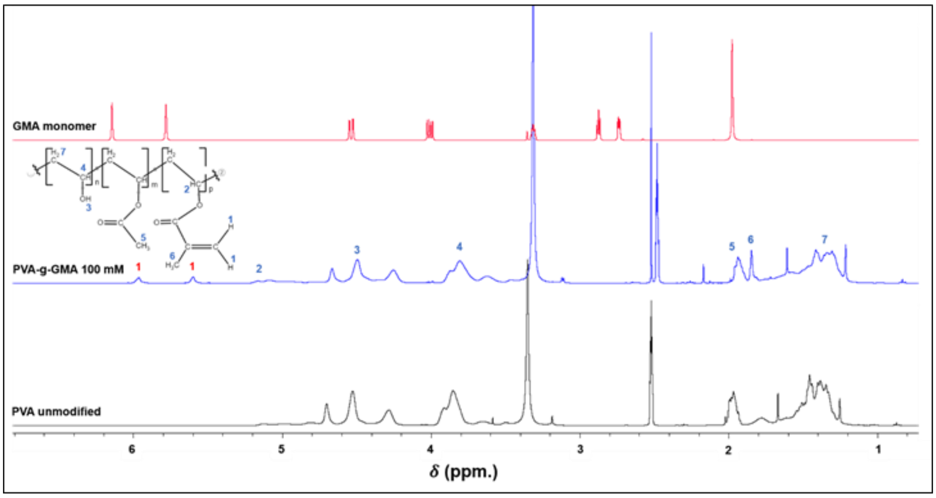

Figure S2. The ^{1}H NMR spectra of GMA monomer, PVA-g-GMA 100 mM, and PVA unmodified.

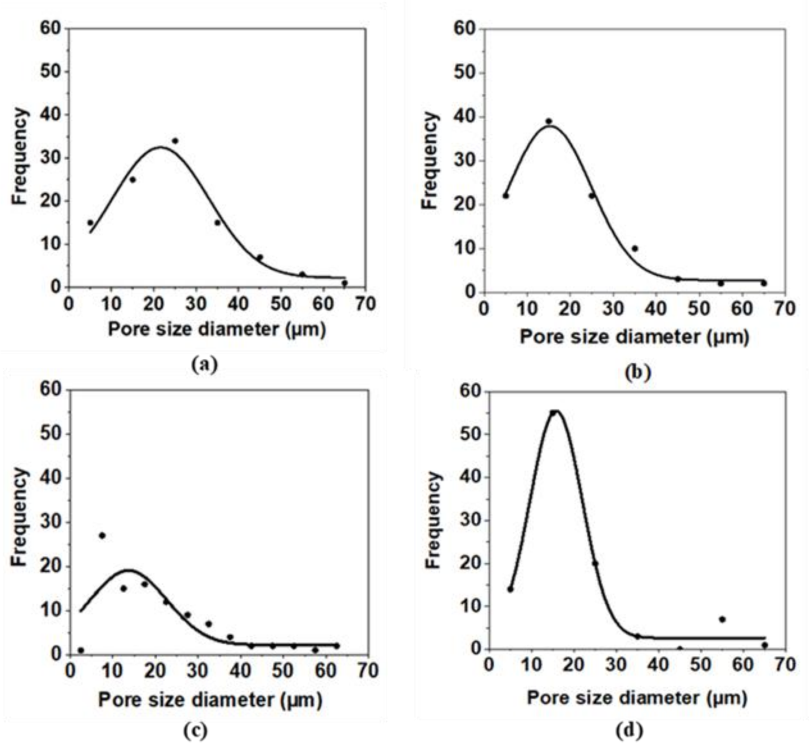

Figure S3. Pore size diameter distribution of (**a**) 10%PVA-g-GMA, (**b**) 10%PVA-g-GMA/0.3%CNF, (**c**) 10%PVA-g-GMA/0.5%CNF, (**d**) 10%PVA-g-GMA/0.7%CNF injectable hydrogel.
